# Brainstem Diffusion Tensor Tractography and Clinical Applications in Pain

**DOI:** 10.3389/fpain.2022.840328

**Published:** 2022-03-24

**Authors:** Yu Zhang, Ansgar J. Furst

**Affiliations:** ^1^War Related Illness and Injury Study Center (WRIISC), VA Palo Alto Health Care System, Palo Alto, CA, United States; ^2^Department of Psychiatry and Behavioral Sciences, Stanford University School of Medicine, Palo Alto, CA, United States; ^3^Department of Neurology and Neurological Sciences, Stanford University School of Medicine, Palo Alto, CA, United States; ^4^Polytrauma System of Care (PSC), VA Palo Alto Health Care System, Palo Alto, CA, United States

**Keywords:** diffusion tensor imaging, diffusion tensor tractography, brainstem, pain, descending pain modulation

## Abstract

The brainstem is one of the most vulnerable brain structures in many neurological conditions, such as pain, sleep problems, autonomic dysfunctions, and neurodegenerative disorders. Diffusion tensor imaging and tractography provide structural details and quantitative measures of brainstem fiber pathways. Until recently, diffusion tensor tractographic studies have mainly focused on whole-brain MRI acquisition. Due to the brainstem's spatial localization, size, and tissue characteristics, and limits of imaging techniques, brainstem diffusion MRI poses particular challenges in tractography. We provide a brief overview on recent advances in diffusion tensor tractography in revealing human pathways connecting the brainstem to the subcortical regions (e.g., basal ganglia, mesolimbic, basal forebrain), and cortical regions. Each of these pathways contains different distributions of fiber tracts from known neurotransmitter-specific nuclei in the brainstem. We compare the brainstem tractographic approaches in literature and our in-lab developed automated brainstem tractography in terms of atlas building, technical advantages, and neuroanatomical implications on neurotransmitter systems. Lastly, we summarize recent investigations of using brainstem tractography as a promising tool in association with pain.

## Introduction

The brainstem is a central structure that connects the brain cerebrum to the spinal cord and cerebellum. The brainstem contains nuclei and fiber pathways that synthesize and transfer specific neurotransmitters and neuromodulators to the peripheral receptors and effectors for the regulation of such basic functions as arousal, motor function, memory, reward, nociception, and autonomic control. Along a dorsoventral axis, the brainstem consists of three subdivisions with distinctive parts: the dorsal side of the midbrain known as the tectum, including the mid-dorsal part of the aqueduct, a central region of nuclei and fibers known as tegmentum beneath the ventricular system, and a massive fiber communication system connecting to the spinal cord and the cerebellum in the ventral part. Nuclei and fiber pathways in the tectum relay pain signals from peripheral stimuli via the spinal cord to the cerebral sensory cortices that result in the sensation of pain (nociception). They also transmit anti-nociceptive signals down to the spinal cord which induces endogenous opioid-based analgesia (pain regulation). Many pain syndromes can be caused by damage to or dysregulation of the brain, brainstem, spinal cord and fibers connecting them. Invasive (surgical) inspection of such damage is often carrying considerable risk which is why non-invasive imaging techniques are a very attractive alternative.

**Figure 1:**
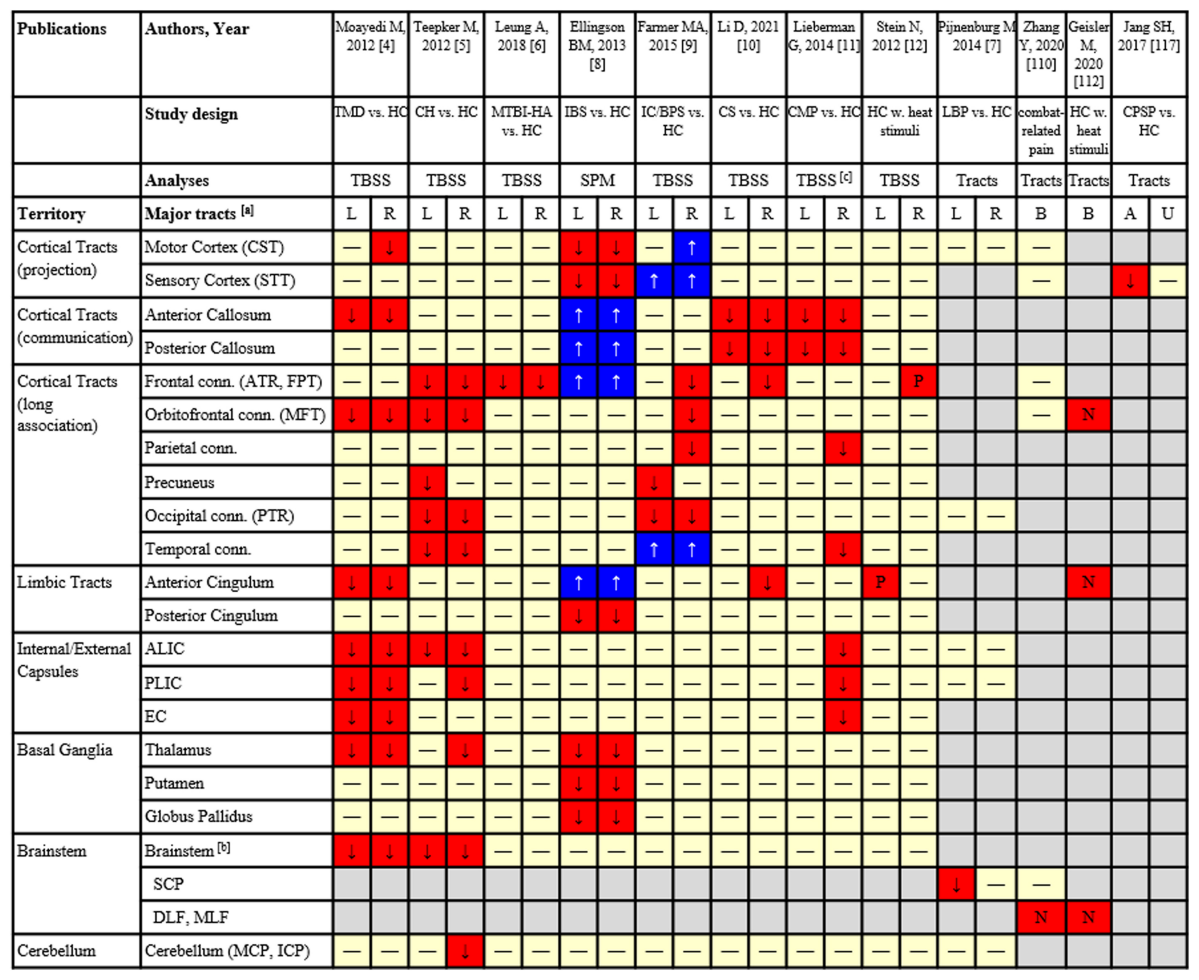
Results from tract-based analyses based on DTI of pain studies: 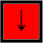 FA significantly reduced in patients; 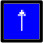 FA significantly increased in patients; 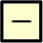 FA was not significantly different; 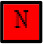 Reduced FA signficantly correlated with increased pain intensity/sensitivity; 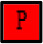 Increased FA significantly correlated with greater analgesic response; 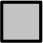 Not analyzed. [a] Cortical and subcortical areas of major fiber tracts (specific tracts included); [b] Some areas in the brainstem without specific definition of tracts. [c] Significant findings are presented based on RD instead of FA which showed a weaker significance. TMD, temporomandibular disorder; HC, healthy control; CH, cluster headache; MTBI, mild traumatic brain injury; IBS, irritable bowel syndrome; IC/BPS, interstitial cystitis/bladder pain syndrome; CS, cervical spondylosis with pain; CMP, chronic musculoskeletal pain; LBP, low back pain; CPSP, central post-stroke pain; TBSS, tract-based spatial statistics; SPM, statistical parametric mapping; CST, corticospinal tract; STT, spinothalamic tract; ATR, anterior thalamic radiation; FPT, frontopontine tract; MFT, medial forebrain tract; PTR, posterior thalamic radiation; ALIC, anterior limb of internal capsule; PLIC, posterior limb of internal capsule; EC, external capsule; SCP, superior cerebellar peduncle; DLF, dorsal longitudinal fasciculus; MLF, medial longitudinal fasciculus; MCP, middle cerebellar peduncle; ICP, inferior cerebellar peduncle.

Conventional brain structural MRI studies have assessed how gray matter volume and white matter integrity are associated with acute pain processing or the severity in chronic pain. Diffusion tensor imaging (DTI) is a specific structural MRI sequence that allows for non-invasive measurement of altered microstructural integrity in particularly white matter regions and tracts in human brain. DTI measures such as fractional anisotropy (FA) explain the strength of the orientational organization of microstructures, radial (RD), axial (AD) and mean (MD) diffusivities are likely to be sensitive to myelin/axon as well as non-specific physiopathology states. In neurological conditions, it is generally believed that reduced FA and increased MD, RD or AD are attributed to demyelination, loss of axons (1, 2), or an interrupted connection (3), which might represent potential damage of the structure in the testing anatomy. Several DTI studies (see Table 1), using non-hypothesis-driven whole brain voxel-wise analysis, found significant microstructural damage in many cerebral regions/fiber connections (some including brainstem fiber tracts) in painful conditions, such as temporomandibular disorders (4), episodic cluster headache (5) or traumatic brain injury-induced chronic headache (6), ankle muscle proprioception in low back pain (12), chronic irritable bowel syndrome (7), chronic pelvic pain (8), cervical spondylosis-induced pain (9), chronic musculoskeletal pain (10), and analgesia in response to pain stimuli (11). These studies have suggested that microstructural damage in the brainstem fiber connections may be associated with pain regulation or pain sensation. Figure 1 indicates the brain tracts that were found to be significantly affected by pain conditions from all published clinical studies reviewed in this article. The majority of these findings were reported in large fiber bundles and brainstem-to-cerebral cortical connections (16).

**Table 1:** Technical details and major findings of the DTI application studies (voxel-based, as well as tract-based) in pain.

**Reference**	**Sample size**	**MRI scanner**	**DTI parameters**	**Analytic approaches**	**Key features of pain**	**Brainstem regions with significant FA reduction / association**	**Other brain regions with significant FA reduction / association**
Moayedi et al. (4)	17 patients vs. 17 HC	3T, GE	b = 0,1 k s/mm^2^, 23 directions, 1.9 x 1.9 x 3 mm^3^ resolution	Non-hypothesis-driven TBSS	TMD-related chronic pain	Trigeminal nerve; brainstem	Internal/external capsules, thalamic and corpus callosum, cingulum
Teepker et al. (5)	7 patients vs. 7 HC	1.5T, Siemens	b = 0,1k, 30 directions, 2 x 2 x 2.4 mm^3^ resolution	Non-hypothesis-driven TBSS	Cluster headache	Brainstem	Frontal, temporal, occipital lobes, internal capsule, thalamus and cerebellum
Leung et al. (6)	10 patients vs. 10 HC	1.5T, GE	b = 0,1k s/mm^2^, 54 directions, 2 x 2 x 2 mm^3^ resolution	Non-hypothesis-driven TBSS	MTBI-induced persistent headache	No significant findings	Left superior longitudinal fasciculus, right anterior thalamic radiation
Ellingson et al. (7)	33 patients vs. 93 HC	3T, Siemens	b = 0,1k s/mm^2^, 61 directions, 3 x 3 x 3 mm^3^ and 2x2x3mm^3^ resolution	Non-hypothesis-driven SPM	Irritable bowel syndrome	No significant findings	Thalamic regions, the basal ganglia and sensory/motor association/integration regions
Farmer et al. (8)	34 patients vs. 32 HC	3T, GE	Not mentioned	Non-hypothesis-driven TBSS	Interstitial cystitis/bladder pain syndrome	No significant findings	Right anterior thalamic radiation, left forceps major, and right longitudinal fasciculus
Li et al. (9)	42 patients vs. 42 HC	3T, GE	b = 0,1k, 30 directions, 2 x 2 x 4 mm^3^ resolution	Non-hypothesis-driven TBSS	Cervical spondylosis-induced pain	No significant findings	Genu, body, and splenium of corpus callosum, and the right anterior corona radiata
Lieberman et al. (10)	46 patients vs. 33 HC	3T, Philips	b = 0,1k, 46 directions, 2 x 2 x 2 mm^3^ resolution	Non-hypothesis-driven TBSS	Chronic musculoskeletal pain	No significant findings	Splenium of corpus callosum, and left cingulum adjacent to the hippocampus
Stein et al. (11)	24 HC	3T, Siemens	b = 0,1k s/mm^2^, 60 directions, 1.7 x 1.7 x 1.7 mm^3^ resolution	Non-hypothesis-driven TBSS	Placebo analgesic responses to thermal stimulation	Periaqueductal gray and connection to the rostral anterior cingulate and prefrontal cortices	Right dorsolateral prefrontal cortex, left rostral anterior cingulate cortex
Pijnenburg et al. (12)	18 Patients vs. 18 HC	3T Philips	b = 0,1.3k s/mm^2^, 60 directions, 2.5 x 2.5 x 2.5 mm^3^ resolution	Hypothesis-driven tract -atlas based	non-specific low back pain	Superior cerebellar peduncle	Not applicable
Zhang et al. (13)	90 patients	3T, GE	b = 0,1k s/mm^2^, 60 directions, 1 x 1 x 2.5 mm^3^	Hypothesis-driven brainstem tract-atlas based	Fibromyalgia-like, chronic pain	Dorsal longitudinal fasciculus	Not applicable
Geisler et al. (14)	38 HC	3T, Siemens	b = 0,1.2k s/mm^2^, 81 directions, 1.7 x 1.7 x 1.7 mm^3^ resolution	Hypothesis-driven manual tractography based	Pain intensity to heat stimuli	Medial forebrain bundle	Not applicable
Jang et al. (15)	5 Patients *vs*. 8 HC	1.5T, Philips	b = 0,1k s/mm2, 32 directions, 1.3 x 1.3 x 2.5 mm^3^ resolution	Hypothesis-driven manual tractography based	Central post-stroke pain	Spinothalamic tract	Not applicable

*HC, healthy control; TMD, temporomandibular disorder; TBSS, tract-based spatial statistics; MTBI, mild traumatic brain injury; SPM, statistical parametric mapping*.

To date, the human brainstem remains one of the most challenging regions to explore with DTI due to the difficulty of accurately delineating small nuclei and complex fiber connections in this area. It is thus necessary to create an atlas of the brainstem small nuclei/fiber tracts in standard neuroimaging space (e.g., in MNI space) (17, 18), and perform imaging analysis by co-registering individual DTI to the MNI space (or vice-versa).

This mini-review is aimed to provide a brief overview on recent advances in brainstem tractography based on DTI, particularly focusing on the technical improvement of automated tractographic approaches. We also discuss the neuroanatomical relevance of brainstem tractography to pain, practical applications and potential implications of brainstem tractography for clinical studies of pain. We hope this article will improve the understanding of the basic principles, help with result interpretation, and increase the appreciation of technical advantages/limitations in DTI tractography. A critical understanding of this technique will promote its use and foster its application in the clinical setting for surgical interventions (19, 20) treatment and evaluation.

## Diffusion Tensor Tractography of the Brainstem

### Manual Tractography

Diffusion tensor tractography offers orientation-based 3-dimensional reconstruction to display neural fiber tracts using data collected from DTI. Specifically, a tractographic algorithm integrates continuous voxel-to-voxel predominant orientations into a fiber streamline that connects distant brain voxels/regions. The initial step of tractography is to fit the non-directional (i.e., b = 0) image and the directional (at least 6) diffusion weighted-images to a tensor model with eigenvectors (i.e., the directions) and eigenvalues (i.e., the strength of diffusions along certain direction). After tensor fitting, tractographic approaches basically use deterministic algorithms (e.g., fiber assignment by continuous tracking – FACT) (21) or the probabilistic algorithms (22) to reconstruct fiber streamlines. These algorithms require placement of anatomical landmarks (region-of-interest, ROI) at one (a single “seed”) or two ends (a pair of “seed” and “target”) of the proposed tract to display pathway between distant brain regions. Deterministic tractography assumes a principal orientation at each voxel, and propagates streamlines from the “seed” voxel to the next voxel with similar principal direction, until stopping criterion (such as a low FA or a sharp angle). The deterministic tractography algorism runs simple, fast, reliable but is sensitive to low directional voxel (e.g., crossing fibers or noise), leading to underestimation/interruption of the streamlines. Probabilistic tractography assumes a distribution of orientations between voxels. It reacts “how likely” each other voxel orientation (according to their likelihood) is to lie along a fiber, and presents the connection likelihood or probability of the streamlines. Probabilistic tractography recovers more extensive fiber bundles but at the price of generating more invalid connections (i.e., overestimation/“false positive” fiber tracts) (23). Despite these limitations, in human medicine, diffusion tensor tractography visualizes anatomical fiber tracts of the most important pathways of the central nervous system, and has been useful in understanding human brain anatomy and surgical planning. Further, the quantitative measurement of fiber tracts provides evaluation of the microstructural status of the myeline membrane, axon bundles, and structural connectivity, and thus has great potential for studying pathological conditions and correlations with clinical symptoms.

A majority of diffusion tensor tractography approaches extract major cerebral fiber tracts that are well myelinated. Brainstem tractography is difficult and requires accurate ROI delineation by neuroanatomical experts because anatomical landmarks are small, fiber networks are short, poor myelinated, joining, and/or crossing over. Manual DTI tractography was used in many studies that aimed to represent the complex anatomy of the brainstem (24–29). Beyond providing normal brainstem anatomy, some other studies focused on specific pathways that have physiopathologic implication, such as the main sensory pathways [e.g., the medial lemniscus, spinothalamic, the cerebropontocerebellar_tracts (30–37), the cranial nerves and their nuclei (38–41), the ascending reticular activating system (ARAS) (24, 42–44), the dopaminergic pathways, (45–48), and the medial forebrain bundle (49–53).

### Automated Tractography and Atlas Building

Manually placing anatomical landmarks is time-consuming and relies on accurate placement of landmarks by neuroanatomical experts. Recent research studies have developed two popular strategies for automated white matter parcellation (54–56), including a white-matter-atlas-based fiber clustering method (57–61) and a landmark-based (through an atlas of gray and white matter ROIs) method (62–70). Both strategies launch individual tractography by transforming an atlas of fiber clusters/landmarks from standard space (e.g., MNI, ICBM, Talairach, etc.) to a subject's native space. In detail: (1) The Fiber clustering strategy provides anatomical fiber tract parcellation (including deep white matter tracts, short and medium range superficial fiber trajectories) by grouping streamlines into clusters on the basis of fiber similarity properties. (2) The landmark-based strategy aims to provide cortical or subcortical parcellations (i.e., ROIs) of the two termini of a fiber tract. Individual tractography can be performed by launching tractography using a “seed” and target ROIs, or extracting fiber streamlines only between the pair of the segmented ROIs from the whole brain tractography. A majority of the landmark-based strategies use the Freesurfer (http://freesurfer.net/) package which provides reliable cortical/subcortical-based-parcellation. However, automated tractography of the brainstem and deep brain areas is limited, due to the lack of an atlas with anatomical landmarks or standardized fiber clusters. Several studies (71–73) provided up to 13 major motor and sensory brainstem tracts using a high-quality high–angular resolution diffusion MRI (HARDI) data from the Human Connectome Project (HCP) via manually-drawn ROI-based (71, 72), or clustering (73) strategies. Furthermore, few studies (71, 74) validated the anatomical precision of brainstem tractography with postmortem or histological sections. Two recent studies (75, 76) also presented similar brainstem tract atlas including motor, sensory, and reticular segments based on routine DTI-MRI sequences. One study built (77) an atlas of the spinal trigeminal tract (SpTV) for studies of trigeminal neuralgia.

Even though these studies (Table 2) of landmarks or fiber clusters in the brainstem are promising, the actual use of these atlases will largely depend on the imaging/IT environment of the end-user. For example, most brainstem fiber atlases have been built based on high-quality, high-resolution, long-duration diffusion scans of healthy young adults, whereas most clinical DTI data are acquired with lower resolution (>2 mm^3^) and a smaller number of directions (≤80) where some thin deep tracts often cannot be successfully visualized. Conventional DTI scans also tend to suffer from susceptibility-induced distortions in the skull-base and the brainstem area, which are difficult to correct. This may lead to an incorrect fiber anatomy in some cases. Furthermore, transforming atlas maps of young healthy brains to groups of individuals with significant changes in brain morphology due to pathological conditions often results in spatial inaccuracies such as misregistration which can ultimately lead to failed or distorted tractographic outputs. In this context, test-retest studies are needed to validate the reliability of brainstem tractographic atlases in the clinical setting. Nonetheless, when performing individual tractography on clinical data using a brainstem atlas, applying sufficient distortion correction (78), data-based customized registration (76), and quality control by neuroanatomical experts in addition to automated processing could improve reliability.

**Table 2:** Summary of studies of brainstem atlas building.

**Reference**	**Sample size**	**DTI parameters**	**Tracking algorithm**	**Tracking approach**	**Brainstem distortion control**	**Parcel lation strategy**	**Brainstem tract atlas**
Meola et al. (71)	488 healthy young adults from HCP	3T, b = 1, 2, 3 k s/mm^2^, 270 directions, 1.25 mm isotropic	DSI Studio	Deterministic tractography	No	Formalin-fixed surgical landmarks of 5 brains	SCP, MCP, ICP, FPT, POTPT, CST, STT, ML, LL, RST, CTT, MLF, DLF
Tang et al. (72)	20 selected healthy young adults from the 488 healthy young adults (HCP)	3T, b = 1, 2, 3 k, 270 directions, 1.25 mm isotropic	FOD-based tractography in MRTrix	Probabilistic tractography	Visual exclusion	Landmarks	FPT, POTPT, ML, STT, LL, SCPCT, SCPCR, SCPSC, MCP, ICPMCT, IVPVCT
Mate et al. (75)	20 healthy subjects	1.5T, b = 1k, 60 directions 2.4 mm isotropic	FMRIB Software Library	Probabilistic tractography	No	Landmarks	Frontopontine, Motor, Sensory, Reticular segments
Yeh et al. (73)	842 healthy young adults from HCP	3T, b = 1, 2, 3 k, 270 directions, 1.25 mm isotropic	DSI Studio	Deterministic tractography	Yes	Clustering	CTT, DLF, LL, ML, MLF, RST, STT
Zhang et al. (76)	62^*^ mid-adult veterans with medical conditions	3T, b = 1 k, 60 directions, 1 x 1 x 2.5 mm^3^	TrackVis	Deterministic tractography	Yes	Landmarks	DLF, MLF, SCP, NST, MFT, CST, STT, FPT, POTPT
Burkett et al. (77)	20 trigeminal patients	3T, b = 1 k, 64 directions 2.0 mm isotropic	StealthViz	Deterministic tractography	Visual exclusion	Landmarks	SpTV
Adil et al. (74)	Postmortem brainstem of a 65-year-old male within 24hr of death	7T, b = 4 k, 120 directions, isotropic resolution of 200 μm^3^	DSI Studio	Deterministic tractography	Not Applicable	Landmarks	AC, CST, DRTSCP, ICP, ML, Facial Nerve, Optic Tracts, PC, TN & SpTV

*HCP, human connectome project; FOD, connectome modeling techniques including fiber orientation distribution; FACT, fiber assignment by continuous tracking; SCP, superior cerebellar peduncle; MCP, middle cerebellar peduncle; ICP, inferior cerebellar peduncle; FPT, frontopontine tract; POTPT, parieto-occipitotemporopontine tracts; CST, corticospinal tract; STT, spinothalamic tract; ML, medial lemniscus; LL, lateral lemniscus; RST, rubrospinal tract; CTT, central tegmental tract; MLF, medial longitudinal fasciculus; DLF, dorsal longitudinal fasciculus; SCPCT, cerebellothalamic tract of SCP; SCPCR, cerebellorubral tract of SCP; SCPSC, spinocerebellar tract of SCP; ICPMCT, ICP from medulla oblongata to the cerebellum; IVPVCT, ICP from the vestibulocerebellar tract; NST, nigrostriatal tract; MFT, medial forebrain tract; SpTV, spinal trigeminal tract; AC, anterior commissure; DRTSCP, dentatorubrothalamic tracts as a subset of SCP; TN & SpTV, trigeminal nerve roots and SpTV*.

* *Our most recent atlas has been updated with an averaged tract map from 62 veterans, from the 17 samples in this publication*.

## Brainstem Circuitry and Pain Modulation

Many experimental and preclinical studies have investigated brainstem small nuclei and circuits that are involved in pain (79), including the periaqueductal gray matter (PAG), rostral ventromedial medulla (RVM), locus coeruleus (LC), dorsal horn (DH) of the spinal cord, and a PAG-RVM-DH circuitry/axis that interconnects them. The PAG sends direct and indirect projection to RVM and DH (80). Part of PAG is involved in an endogenous analgesia, as direct stimulation of the PAG can suppress pain intensity in individuals with various chronic pain conditions (81–83). Numerous studies have suggested a potential mechanism of descending pain inhibition and its pathways through the PAG-RVM-DH axis (84–88). In contrast to the role of reducing pain, some experimental studies have suggested that excitation of the PAG-RVM-DH system can lead to a facilitating effect through the DH (89, 90), SpV (91), and RVM (92) resulting in hyperalgesia.

To extend our understanding of the PAG-RVM-DH pathways gained through experimental studies in animals, human brain functional MRI (fMRI) studies have been promoted to investigate brainstem functional activation patterns associated with pain sensitivity and pain modulation. An extensive number of fMRI studies have reported the brainstem being involved in descending analgesic responses in various pain conditions. For example, a resting state-fMRI study (93) reported patients with chronic orofacial pain show increased functional connectivity between the RVM, LC and PAG, as well as supratentorial regions (e.g., hippocampus, nucleus accumbens, and anterior cingulate cortex), possibly reflecting “top–down” engagement of the circuitry alongside altered reward processing in pain conditions. Other studies found that higher functional connectivity between PAG and RVM (or LC) is associated with higher pain scores in fibromyalgia (94), as well as in chronic neuropathic orofacial pain (93) and diabetic polyneuropathy-induced pain (95). Several studies (96, 97) investigated healthy volunteers during heat stimulation and distraction (e.g., participants were asked to perform a color-word Stroop distractor task while receiving thermal stimuli) and found that distraction was associated with a significant reduction of pain intensity with increased functional activation of the cingulate, hippocampus, thalamus, PAG, and brainstem regions, exerting a “top-down” influence on pain modulation during distraction. In addition to the PAG-RVM-DH pathways, several fMRI studies also revealed that greater functional connectivity of the medial prefrontal cortices to the amygdala and nucleus accumbens are associated with severer acute (98) and chronic pain (99–101).

## Brainstem Tractography and Relevance to Pain

The neuroanatomical and neuropsychological studies of pain (102) suggest a complex network comprised of ascending and descending pathways in the brain. The descending pathway, also known as the “top down” pathway via the PAG-RVM-DH axis, has been demonstrated to be an endogenous analgesic system that is activated in response to pain stimuli. The ascending pathways transmit nociceptive signals from peripheral nerves to the sensory cortex via the dorsal horn of the spinal cord, brainstem, and thalamus (103). While these complex neurotransmitter pathways can be represented through tractography, our understanding of the anatomical connectivity and its implication in pain processing or pain control is still an emerging field of exploration.

**Figure 2:**
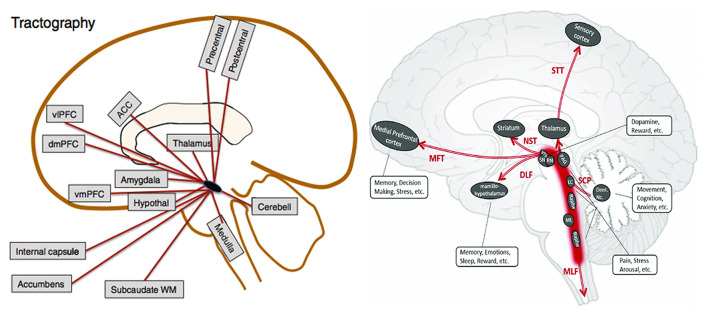
Possible PAG connections based on DTI from Linnman et al. (84) and Zhang et al. (76). ACC, anterior cingulate cortex; Cerebell, cerebellum; dmPFC, dorsomedial prefrontal cortex; vmPFC, ventromedial prefrontal cortex; vlPFC, ventrolateral prefrontal cortex; WM, white matter; VTA, ventral tegmental area; SN, substantia nigra; RN, red nucleus; PAG, periaqueductal gray matter; LC, locus coeruleus; ML, medial lemniscus; Dent. Nc., dentate nucleus; STT, spinothalamic tract; NST, nigrostriatal tract; MFT, medial forebrain tract; DLF, dorsal longitudinal fasciculus; SCP, superior cerebellar peduncle; MLF, medial longitudinal fasciculus.

Recent improvements in diffusion tensor tractography allow presentation of major brainstem pathways in humans. Given its role in pain modulation several studies performed diffusion tractography of the PAG manually (104) or a PAG site used in deep brain stimulation (DBS) (105–107) for treatment of chronic pain to identify fiber tracts that might be associated with pain. Tracts that are consistently presented in these studies include: (1) PAG to the thalamus, which overlaps with the superior cerebellar peduncle (SCP) and the spinothalamic tract (STT); (2) PAG to the medial prefrontal cortex through ventral tegmental area (VTA) and nucleus accumbens, which overlaps with the medial forebrain tract (MFT); (3) PAG to the hypothalamus and RVM, which overlaps with the dorsal longitudinal fasciculus (DLF) and partially the medial longitudinal fasciculus (MLF). Further anatomical connections with the PAG beyond the brainstem area include amygdala, anterior cingulate cortex, ventromedial prefrontal cortex (which overlaps with MFT), ventral posterior thalamus and primary somatosensory cortex (which overlaps with STT). A schematic map of the tracts that possibly relate to pain displayed in Figure 2.

**Figure 3:**
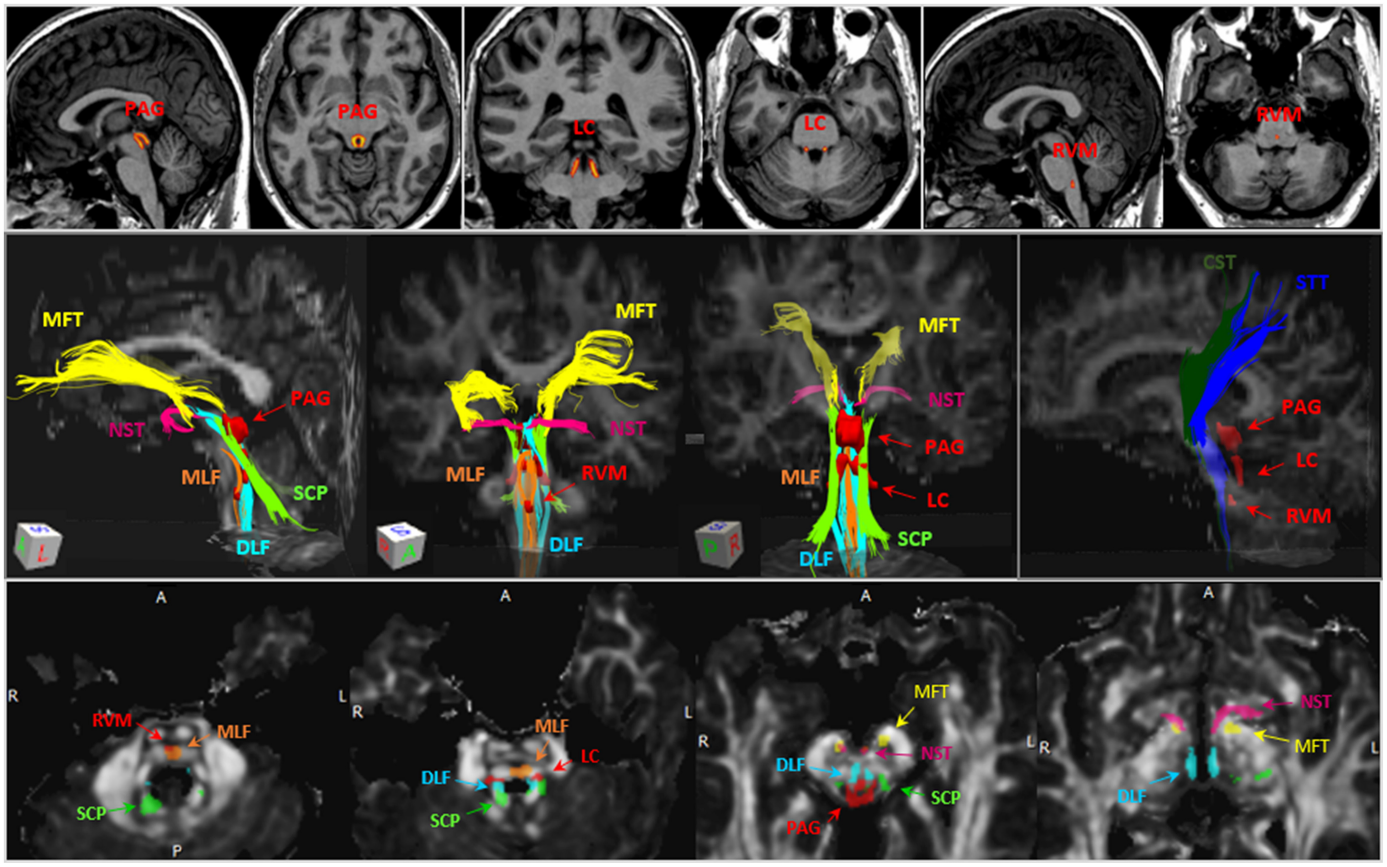
Illustration of ROI where the volumes of the brainstem nuclei and diffusion metrics were measured. *Upper panel*, Illustration of ROIs of the PAG, LC and RVM, which were delineated based on literature (108). The hot color scale represents probability of gray matter density (brighter color refers to higher gray matter density) within the ROIs. *Middle panel*, example of brainstem tracts of interests, including MLF (orange), DLF (cyan), SCP (green), NST (dark pink), MFT (yellow), CST (dark green), STT (blue), and the three brainstem nuclei (red). *Lower panel*, the anatomical relationships between brainstem tracts (non-red) and nuclei (red) on 4 brainstem axial slices. PAG, periaqueductal gray; LC, locus coeruleus; RVM, rostral ventromedial medulla; MLF, medial longitudinal fasciculus; DLF, dorsal longitudinal fasciculus; SCP, superior cerebellar peduncle; NST, nigrostriatal tract; MFT, medial forebrain tract; CST, corticospinal tract; STT, spinothalamic tract.

On the basis of existing automated brainstem DTI tractographic atlases, Figure 3 presents an illustration of the fiber tracts that are highly relevant to pain and their relationship with key brainstem nuclei (e.g., PAG, LC and RVM). The anatomy of these brainstem tracts can be confirmed with known neuroanatomical books (108–111).

The DLF connects hypothalamus, PAG and spinal cord via the ventral side of the aqueduct and fourth ventricle. The DLF anatomy is consistent with the inferomedial branch of the medial forebrain bundle that has been described in several DBS studies (112), which consists of fiber that connects through upper pons, retrorubral area, PAG, VTA in the midbrain and ends in the lateral hypothalamus. Using atlas-based automated brainstem tractography (76), our recent study reported that FA decrease and MD/RD/AD increase in the DLF are associated with increased pain intensity in a group of patients with fibromyalgia-like, chronic pain (13). The DLF appears to have the largest anatomical overlap with the described descending analgesic pathway which in turn suggests that damage of the DLF may result in impaired endogenous pain regulation.

The SCP connects the dentate nucleus with the thalamus, red nucleus, vestibular nuclei, and the reticular formation. It functions mainly as a network for motor coordination and postural control. The SCP at the midbrain level includes non-decussated and decussated branches that partly overlap with PAG and VTA (113). One study (12) using an automated atlas-based approach, reported FA decrease and MD increase of the SCP to be significantly associated with greater sensation of low back pain in response to ankle muscle vibration in healthy volunteers.

The MFT connects between deep cerebellar nuclei and anterior frontal regions. It traverses partially through the VTA, PAG, inferomedial thalamus, also passes through the anterior limb of internal capsule (ALIC) along with anterior thalamic radiation (ATR). Similar MFT connection, which is described as a superolateral branch of the medial forebrain bundle in a previous review article, is considered to be a seeking/pleasure pathway (112). DBS treatment on this pathway showed anti-depressive effects and modified the emotional pain state, suggesting that the MFT may have therapeutical implication for affective pain. Two studies investigated MFT (or fibers with similar anatomy) changes in response to pain in healthy subjects. One using a semi-automated approach (which manually delineates tractographic landmarks from the FreeSurfer parcellation atlas), reported that FA reduction and RD increase in the MFT are associated with higher pain sensitivity after heating stimuli (14). Another study, using a voxel-wise statistical approach with a FA-based tract skeleton, showed reduced FA in several tracts (connecting dorsolateral prefrontal cortex, rostral anterior cingulate cortex with the PAG) was associated with better placebo analgesic responses (11), which supports the notion that lower FA in the MFT is associated with decreased sensitivity to painful stimuli.

The STT is known as a major sensory pathway that is highly trackable and reproducible by either manual or automated approaches. The STT consists of a spinothalamocortical pathway (pain and temperature sensation) and a medial lemniscothalamocortical pathway (conscious proprioception) (114). Both pathways are considered the main ascending sensory pathways of the body that travel rostrally in close proximity within the brainstem to the thalamus and to the somatosensory cortex (115). Several papers showed central post-stroke pain (those without signs of peripheral neuropathy) was related to injury of the STT detected by loss of the STT volume (116), thickness (117), or damaged microstructural integrity including FA decrease and MD increase (15).

Taken together, these studies applying diffusion tensor tractography in pain research demonstrated that some dorsal brainstem fibers may be involved in pain processing and modulation on the basis of these fiber tracts connecting the cerebral cortices through the PAG to the dorsal spinal cord. These studies consistently observed a significant correlation between a reduced microstructural integrity of the brainstem tracts connecting to PAG and increased pain intensity/sensitivity. These findings suggest a structural connectivity-related endogenous pain regulation. Specifically, substantial impairment (e.g., impaired axon or myeline membrane) of the brainstem fiber connection results in a dysfunction of the descending inhibition or modulation. Although the clinical application of diffusion tensor tractography in pain is still infrequent, the above evidence suggests that its inclusion in future studies may hold a lot of promise.

## Limitations

Current pain research applying brainstem diffusion tensor tractography has been limited in several ways: (1) Most of the brainstem tractography atlases have been built based on healthy young adults with sophisticated diffusion protocols that would last longer than 50 min. A super-high resolution (e.g., 200 Mm isotropic voxel) using higher magnetic field strengths such as 7T scanner could further improve tractographic atlas building and analyses in microstructural level but 7T scanners are typically not available in hospitals as they provide limited clinical relevance. This is further complicated by the fact that atlases build on 7T data will not directly translate into lower field scans like the commonly used 3T clinical scanners. Conversely, clinical DTI protocols routinely tend to be no longer than 10 min with lower resolution (≤2 mm^3^) and fewer diffusion directions (≤80). Therefore, in clinical practice, small pathways may not be identifiable and mis-registration may often occur in patients with conditions affecting brain morphology including elderly patients. Further technical developments should be focused on improvement of quality and feasibility of clinical scans, as well as correcting errors due to low resolution, noise, artifacts, distortion and crossing-fibers during post-processing. (2) Although a number of existing diffusion tensor tractographic studies have consistently attributed a role to the structural connectivity of brainstem tracts in descending analgesic pathways, less is known whether these tracts might also be associated with ascending pain facilitation effects. It is technically even difficult because the diffusion tensor tractography expresses a bidirectional structure that cannot separate the afferent or efferent fiber inputs, thus cannot distinguish the ascending or descending pathways, respectively. (3) Generally, alterations of quantitative DTI variables explain non-specific biological features and therefore only permit indirect interpretations of the underlying pathophysiology. The important role of brainstem fibers was discussed here together with supporting evidence. Additional pathophysiologic validation is needed to further understand the role of brainstem fibers in pain and other conditions.

## Conclusion

As a non-invasive, clinically feasible MRI sequence, DTI is a sensitive imaging method in detecting problems of structural integrity of human brain neural pathways. Recent developments in diffusion tensor tractography offer visualization and quantitative analysis of the status of small brainstem fiber tracts, in which several known pain-related neural pathways are included. A better understanding of the relationship between brainstem structural connection and chronic pain and/or pain modulation will be helpful for revealing the neurobiological basis and regulation mechanisms underlying pain.

## Author Contributions

YZ and AF contributed conception and design of the review. YZ wrote the first draft of the manuscript. AF reviewed the first draft. All authors contributed to manuscript revision, read, and approved the submitted version.

## Funding

This work was funded by the War Related Illness and Injury Study Center (WRIISC) of the Palo Alto Veterans Affairs Health Care System, Department of Veterans Affairs Office of Academic Affiliations and VA Clinical Science Research and Development (CSR&D) grant entitled the role of the brain stem in GWVI pathology (1 I01 CX002182-01).

## Conflict of Interest

The authors declare that the research was conducted in the absence of any commercial or financial relationships that could be construed as a potential conflict of interest.

## Publisher's Note

All claims expressed in this article are solely those of the authors and do not necessarily represent those of their affiliated organizations, or those of the publisher, the editors and the reviewers. Any product that may be evaluated in this article, or claim that may be made by its manufacturer, is not guaranteed or endorsed by the publisher.
